# Associations Between Anthropometric Characteristics, Self-Reported Musculoskeletal and Visceral Symptoms, and Squat Movement Quality: A Cross-Section Study

**DOI:** 10.3390/jfmk11010086

**Published:** 2026-02-20

**Authors:** John Xerri de Caro, Andrew Pirotta, Emanuel Schembri, Malcolm Borg

**Affiliations:** 1 Department of Physiotherapy, Faculty of Health Sciences, University of Malta, MSD 2090 Msida, Malta; esche05@um.edu.mt; 2 UN1Q Fitness, BKR 9037 B’Kara, Malta; andrew@un1q.com (A.P.); malcolm@un1q.com (M.B.)

**Keywords:** squat mechanics, body composition, posture, anthropometry, movement analysis, biomechanics

## Abstract

**Background**: This study investigated associations between anthropometric characteristics, postural deviations, musculoskeletal and visceral symptoms, and squat movement quality to clarify how individual physical attributes and symptom profiles influence fundamental movement performance. **Method(s)**: A cross-sectional observational study recruited adults aged 18–65 who could ambulate without pain. Anthropometric and body composition measures were collected. Standardized posture images and multi-angle squat videos were obtained, and visual classifications of posture and squat technique were conducted using predefined criteria. Descriptive statistics characterized the sample, and multivariable logistic regression with LASSO regularization examined associations between demographic, postural, and symptom variables and binary squat outcomes. **Results**: Two hundred participants (57.5% female; median age 26 years) were included. Males showed higher stature, lean mass, and waist circumference, whereas females exhibited higher body fat and reported more neck pain and headaches. Forward head posture was common (62%), while women demonstrated more favorable upper-body alignment. Most participants maintained neutral lumbar posture and grounded heels during squats, with sex differences in foot rotation and knee path. Higher fat mass predicted reduced squat depth (OR = 1.06, 95% CI: 1.00 to 1.11, *p* = 0.033); heel lift and absent forward knee movement were associated with better spinal neutrality (OR = 0.07 and 0.18, both *p* ≤ 0.002); and low skeletal muscle mass (OR = 0.87, 95% CI: 0.79 to 0.95, *p* = 0.004) and heel lift (OR = 7.09, 95% CI: 1.86 to 26.2, *p* = 0.003) predicted suboptimal knee tracking. Only 8% achieved a fully “perfect” squat. **Conclusion(s)**: Suboptimal squat mechanics were linked to higher fat mass, lower skeletal muscle mass, and compensatory lower-limb strategies, suggesting that squat quality reflects an interaction among body composition, posture, and motor control rather than any single demographic or anthropometric factor.

## 1. Introduction

The relationship between visual classification of squat movements and personal characteristics such as age, sex, race or ethnicity, body mass index (BMI), fat-free mass, and skeletal muscle mass is an important area of research. Maintaining proper static posture is crucial when performing squats, as improper technique can lead to increased injury risk [1]. A wide array of research has demonstrated that upright postural alignment, pelvic positioning, and spinal alignment significantly influence squatting techniques, balance, and ultimately, outcomes during squat exercises. Symptoms such as neck pain, low back pain (LBP), headaches, or hypertension, as well as a history of hernia and urinary incontinence, could also have significant implications for squat mechanics and performance.

While the literature traditionally emphasizes biomechanical factors for squat movements in training and rehabilitation, the interactions among demographic factors, postural factors and self-reported symptoms in influencing squat mechanics do not. Understanding any interactions may be important for optimizing training and rehabilitation strategies for individuals experiencing various physical challenges. While the literature largely focuses on biomechanical changes leading to enhanced physical fitness outcomes, it also stresses the necessity of understanding how inherent body characteristics impact squat performance and visual classification. For instance, variations in body composition among athletes introduce distinct kinematic and kinetic profiles during squats [2], underlining the need for tailored training programs based on visual analyses that consider individual anthropometric profiles. Consequently, both qualitative and quantitative assessments of squat movements can be vital tools for identifying personal characteristics and optimizing training regimens. Visual classification methods including computer vision techniques have been shown to identify specific movement patterns in squatting exercises effectively [3]. This approach can be linked with individual physical characteristics, offering insights into anthropometric data like BMI and body composition [4]. Proper squat mechanics often reflect an individual’s strength and body composition; those with higher muscle mass and lower fat percentages typically display more efficient squat mechanics which correlate to better jump ability [5] and improved muscle cross-sectional area and decreased intramuscular fat [6]. This suggests that improvements in squat performance are linked to physiological changes such as increases in muscle mass and reductions in fat mass, unlike elastic resistance training [7]. Furthermore, personal characteristics like BMI and muscle mass can influence visual assessments of squat performance. Research demonstrates that fat percentage is inversely associated with squat performance in females [8], possibly as it influences an individual’s ability to perform repetitions prior to failure that is relative to anthropometric differences and signals a multifaceted relationship between body composition and repeated squat capacity [9]. This indicates a bidirectional relationship, where each aspect informs the other, allowing visual classification of squat movements to capture the nuances of personal physique that affect performance both biomechanically and physiologically.

While the biomechanical aspects of squat movement analysis are well documented, any reciprocal relationship between visual classification and individual physical characteristics may suggest significant potential for further research to explore how these fields can mutually enhance outcomes related to health and fitness.

Understanding the interactions across demographic factors, postural factors, and self-reported symptoms on squat mechanics may leverage to optimize training and rehabilitation strategies for individuals experiencing various physical challenges. Demographic factors, such as age, sex, and BMI, interact with postural elements to shape squat mechanics - individuals with higher BMI often exhibit postural deviations, such as anterior pelvic tilt, which can obstruct the ability to achieve deeper squats [8,10]. Age-related changes in musculoskeletal function also affect how individuals with varying demographic backgrounds utilize their bodies during squats. Older adults may rely on adaptive movements that can negatively impact their postural alignment, leading to compromised squat mechanics. Studies indicate that rehabilitation programs tailored to account for these demographic and postural interactions can lead to improved outcomes [10,11]. Given that self-reported symptoms like neck or low back pain can differentially influence squat mechanics based on demographic variables such as sex and BMI, research suggests that women, who may report higher pain sensitivity, often adopt greater compensatory movement strategies during the squat [12,13]. Furthermore, those with higher BMI may experience more pronounced pain responses, thereby altering their squat performance significantly compared to those with lower BMI [14]. This highlights the need for comprehensive assessments that consider both self-reported symptoms and demographic context to develop effective strategies for managing squat performance in patients with pain conditions [15]. The combination of demographic factors, postural alignment, and symptom-related variables creates a multifaceted picture that can predict deviations in squat mechanics. Profiles that include high BMI and evident postural misalignments (e.g., increased lumbar lordosis) in the presence of self-reported symptoms such as chronic low back pain can signal significant deviations in squat depth and control [16,17]. Integrating demographic data with robust postural assessments and symptom questionnaires can provide predictive insights for practitioners to tailor interventions effectively [18,19].

Although the independent effects of body composition, posture, and pain on squat biomechanics have been examined, few studies have evaluated their combined multivariable influence on visually assessed squat quality in applied screening contexts. Given that clinical and fitness-based squat assessment relies predominantly on observational classification rather than laboratory motion capture, understanding how anthropometric, postural, and symptom-related factors interact to influence observable movement deviations is essential. This study therefore aimed to investigate the multivariable associations between body composition, posture, symptom profiles, and squat movement quality to inform both clinical screening and the development of camera-based movement analysis systems. We hypothesized that higher fat mass, lower skeletal muscle mass, postural deviations, and the presence of musculoskeletal symptoms would independently predict suboptimal squat mechanics across defined visual performance domains. This study forms part of the STEPS (Screening Technology Enhancement for Posture & Movement through Smartphones) project, funded under Xjenza Malta’s FUSION Research and Innovation Programme (LITE 2024–2025: R&I-2024-017L), which aims to develop smartphone-based movement analysis tools for identifying postural and mobility deficits.

## 2. Materials and Methods

### 2.1. Study Design and Participant Characteristics

To reach the aims of this study, a cross-sectional observational design was adopted to investigate any associations between the quality of the squat movement and personal characteristics of posture, BMI, hip and waist circumference, fat-free mass, and skeletal muscle mass, and self-reported musculoskeletal and visceral symptoms. An optimal (“perfect”) squat was defined as the absence of compensatory movements across all assessed domains, including spinal alignment, knee tracking, foot positioning, depth, and head–neck posture. The following indicators were assessed: (1) anthropometric and body composition measures, (2) static postural characteristics, (3) squat movement quality parameters, and (4) self-reported musculoskeletal and visceral symptoms. These indicators were selected due to their frequent use in clinical movement screening and their relevance for automated visual classification in applied settings. Participants were recruited via convenience sampling and an intense social media campaign that targeted different groups; they had to be aged between 18 and 65, and able to ambulate freely without pain. Prior experience in performing resistance training or squat experience was not necessary. Posture classifications (e.g., thoracic kyphosis, protracted shoulders) were treated as predictor variables, not exclusions. Postural deviations were not excluded but were intentionally retained as independent variables due to their potential influence on squat execution.

### 2.2. Study Protocol and Procedures

The entire study followed a strict research protocol that was examined by the research and ethics board and submitted with the project documentation. Each participant was assigned a unique participant ID and introduced to the study protocol and gave signed informed consent. They proceeded to have still images of their natural posture captured from the side, front and rear using hand-held standard digital RGB-D cameras positioned approximately 1.0m away. They then underwent weight and height measurements followed by body fat measurements using skin calipers. At this point participants laid down on a couch for 5 min in anticipation of Body Composition Analysis (BCA) using the SECA mBCA 525 Bioimpedance Analyser. This was done to give their body time to rest. In the meantime, baseline questions were asked about their general activity and any self-reported visceral symptoms including history of neck pain, low back pain, headaches, hypertension, heartburn, constipation, hernia and incontinence. Following the BCA, they then performed a rigid protocol of several exercises, including the squat exercise, under the guidance and supervision of a physiotherapist. During the execution of the exercises, video recordings were obtained using the same standard digital RGB-D cameras now positioned on a fixed stand at 1.5m from the participant at hip height, capturing frontal and sagittal views. No reflective markers or automated motion analysis systems were used. The physiotherapists were tasked to classify the still images of posture and squat performance was visually assessed during the descent, bottom, and ascent phases, against a pre-assigned classification that was prepared by the research team and that included detailed descriptions of proper technique and faults. While we recognize that there is no single universally accepted, evidence-based “perfect” posture, we defined good posture as neutral sagittal and frontal plane alignment without visible excessive kyphosis, lordosis, forward head displacement, or shoulder protraction consistent with clinical consensus criteria [20,21].

### 2.3. Ethical Considerations

Data confidentiality and anonymity were strictly maintained. All participants were allocated a unique study identification number which was saved with their image and video data. All data were stored securely in encrypted formats on a password-protected computer. Demographic details linking the images and video with a specific person were not stored together with the image and video data. All participants were provided with information on the ways that the data were used, stored and protected during and after the project before providing explicit consent for use of the data as intended. To ensure data security after editing and labeling, the data were stored exclusively in secure online storage servers and were deleted from any laptop or desktop devices. Data that may lead to the identification of participants were also stored offline on an encrypted external hard drive or flash drive and kept in a locked secure place when not in use, with the appropriate access settings applied. Signed written consent was obtained with participants having the option to withdraw at any time. The study complied with the General Data Protection Regulation (GDPR), ensuring that participants could access, rectify, or request the deletion of their data. All participants were treated equally, with equal opportunities to participate in the study. The study received ethical approval from the University of Malta Faculty Research Ethics Committee (FREC) under reference number FHS-2024-00690, ensuring compliance with institutional ethical standards. Participants were offered a financial incentive of €15 each for their contribution.

### 2.4. Data Analysis

Descriptive analyses were conducted to characterize participant demographics and squat mechanics. Continuous variables were assessed for normality using the Shapiro–Wilk test. Missing data were handled using median imputation for continuous variables and mode imputation for categorical data. Associations between demographic, standing posture predictors and binary squat outcomes (e.g., non-neutral spine, heel lift, incorrect knee path) were examined using multivariable binary logistic regression. Given the exploratory nature of the study, a penalized logistic regression (LASSO), with 10-fold cross-validation, was first applied to reduce dimensionality and identify variables showing any evidence of association with the outcome. The variables retained from this screening step were then further refined using backward elimination based on likelihood ratio tests, continuing until only statistically significant predictors remained. Model fit and assumptions were evaluated through likelihood ratio tests, residual diagnostics, and goodness-of-fit indices, including Akaike’s Information Criterion (AIC), pseudo-R^2^, and the area under the receiver operating characteristic curve (AUC). Due to complete or quasi-complete separation in the “perfect squat” outcome, penalized likelihood estimation using Firth logistic regression was applied. Odds ratios with 95% confidence intervals were reported, and statistical significance was set a priori at α = 0.05 (two-tailed). All analyses were conducted in R (version 4.4.0) using the tidyverse, glmnet, logistf, and related packages.

## 3. Results

### 3.1. Participant Characteristics

Participant characteristics are presented in Table 1, Table 2 and Table 3. Of the 200 participants included in the analysis, 57.5% (n = 115) were female and 42.5% (n = 85) were male. The median age of the overall cohort was 26 years (IQR 21 to 35). A total of 91.0% (n = 182) of the participants identified their racial ethnicity as White. There were equal distributions for gender differences in age (F: median age 26, IQR 21 to 38; M: median age 26, IQR 21 to 34) and for those identified as White race (F: n = 105, 91.3%; M: n = 77, 90.6%). Anthropometric characteristics differed significantly by gender with males being taller (mean height 175.3 cm vs. 163.6 cm, *p* < 0.001), heavier (median weight 77.2 kg vs. 61.1 kg, *p* < 0.001), having a greater waist circumference (median circumference 85.0 cm vs. 74.0 cm, *p* < 0.001), lower % fat mass (median 16.0% vs. 26.8%, *p* < 0.001), greater fat-free mass (median 64.2 kg vs. 44.0 kg, *p* < 0.001), and greater skeletal muscle mass (median 32.2 kg vs. 21.0 kg, *p* < 0.001).

The demographic characteristics for 200 participants were assessed across gender against self-reported symptoms (Table 1), posture (Table 2) and squat characteristics (Table 3). For posture the following were considered: head, cervical spine, shoulders, thoracic spine, lumbar spine and pelvic tilt. For self-reported symptoms the following were considered: low back pain, headaches, hypertension, heartburn, constipation, history of hernia and incontinence. For squat characteristics the following were considered: spine movements (butt-wink, lumbar hyperextension and neutral), neck movements (cervical hyperextension and neutral), feet (heel grounded and heel lift), range of movement (ROM) (good depth and lacking depth), knees (forward knees and no forward knees), feet position (less than shoulder width, more than shoulder width and shoulder width), feet rotation (excessive outward rotation, inward rotation and slight outward rotation) and knee path (drive out in line with feet and incorrect knee path).

### 3.2. Self-Reported Symptoms

Low back pain was the most prevalent self-reported symptom (n = 66, 33.0%) followed by neck pain (n = 49, 24.5%) and headache (n = 33, 16.5%). The across gender analysis revealed that neck pain (33.0% vs. 12.9%, *p* = 0.001) and headache (25.2% vs. 4.7%, *p* < 0.001) were significantly more prevalent in women, with low back pain, although the prevalence was higher in women, being borderline non-significant when compared to men (38.3% vs. 25.9%, *p* = 0.066). Other health-related symptoms were less frequently reported with heartburn at 9% (female: male, 13.9% vs. 2.4%, *p* = 0.005) and constipation at 9% (female: male, 13.0% vs. 3.5%, *p* = 0.020); history of hernia (3.5%), hypertension (2.5%) and incontinence (1.5%) showed similar patterns across gender.

### 3.3. Posture

A pronounced forward head posture was present in 61.9% of participants without gender differences (*p* = 0.32). Cervical spine posture was comparable between genders (overall 62.9%; *p* = 0.72). For shoulder and thoracic posture, women were better compared to men with recorded good shoulders (74.1% vs. 48.2%, *p* < 0.001) and good thoracic postures (75.5% vs. 32.9%, *p* < 0.001). Differences across gender for lumbar spine posture (*p* = 0.40) and pelvic tilt (*p* = 0.57) were not noted.

### 3.4. Squat Mechanics

Most participants maintained neutral lumbar alignment (53.0%) and kept heels grounded (92.5%), with no differences being reported across gender. Foot rotation differed by gender (*p* = 0.002), with excessive outward rotation more common in men (43.5% vs. 25.2%). Knee path alignment also differed (*p* = 0.047), with incorrect knee path more frequent in women (14.8% vs. 5.9%). Squat depth, forward knee translation, and stance width did not differ by gender.

These descriptive findings informed the subsequent statistical modeling of eight key squat mechanics negative outcomes (see Appendix A) with the intention to identify any associations leading to poor form.

1. Predictors of Cervical Hyperextension

Cervical hyperextension was recorded in 45.5% (n = 91) of participants. The multivariable model showed acceptable discrimination (AUC = 0.658) and explained only 9.5% of variance (Nagelkerke R^2^). Greater hip circumference was associated with lower odds of cervical hyperextension (OR = 0.96, 95% CI: 0.94 to 0.99, *p* = 0.009). Performing the squat without forward knee was associated with lower odds compared with forward knee translation (OR = 0.49, 95% CI: 0.27 to 0.90, *p* = 0.021), indicating that lower hip circumference and forward knee displacement relate to a greater likelihood of cervical extension.

2. Predictors of Non-Neutral Spine

Movements of the spine during the squat were categorized into two groups, neutral (53.0%) or non-neutral (47.0%), with the latter comprising butt-wink (40.0%) or lumbar hyperextension (7.0%). The model showed good discrimination (AUC = 0.787) and explained up to 31.7% of variance (Nagelkerke R^2^). Higher % fat mass calculated by BCA was associated with lower odds of non-neutral spinal movements (OR = 0.90, 95% CI: 0.86 to 0.94, *p* < 0.001), whereas greater skinfold thickness at Caliper Sites 2 and 3 was positively associated with the outcome (OR = 1.04 for both, *p* = 0.041 and *p* = 0.032, respectively). Heel lift and the absence of forward knee translation were strongly associated with lower odds of non-neutral spine (OR = 0.07 and 0.18, both *p* ≤ 0.002), highlighting the contributions of lower-limb mechanics and anthropometry to spinal alignment during the squat.

3. Predictors of Lacking Squat Depth (ROM)

The range of squat movement was recorded as being good depth (75.0%) or lacking depth (25.0%). Model discrimination was good (AUC = 0.816), and it explained 34.5% of the variance (Nagelkerke R^2^). Each increase in Caliper Site 2 thickness (OR = 1.06, 95% CI: 1.02 to 1.11, *p* = 0.006) and total fat mass (kg) (OR = 1.06, 95% CI: 1.00 to 1.11, *p* = 0.033) was positively associated with a lack of squat depth. A protracted shoulder posture increased the odds of limited depth compared to good shoulder alignment (OR = 2.66, 95% CI: 1.16 to 6.31, *p* = 0.022). Relative to butt-wink, lumbar hyperextension (OR = 24.1, 95% CI: 4.92 to 135, *p* < 0.001) and neutral spine (OR = 5.33, 95% CI: 2.07 to 15.4, *p* = 0.001) both markedly increased the odds of lacking squat depth. Incorrect knee path (OR = 5.53, 95% CI: 1.80 to 17.6, *p* = 0.003) and the absence of forward knee translation (OR = 4.19, 95% CI: 1.70 to 11.5, *p* = 0.003) were also significant predictors.

4. Predictors of Forward Knees

The movements of the knees were recorded as no forward knees (64.5%) and forward knees (35.5) during the squat. The model’s discrimination was good (AUC = 0.784) and explained up to 30.4% of the variance (Nagelkerke R^2^). Higher FM BCA% increased the odds for forward knee translation (OR = 1.05, 95% CI: 1.01 to 1.09, *p* = 0.010), as did low back pain (OR = 2.04, 95% CI: 1.01 to 4.18, *p* = 0.047). Compared to butt-wink, lumbar hyperextension increased the odds of forward knee translation (OR = 4.44, 95% CI: 1.17 to 19.7, *p* = 0.036), whereas neutral spine was associated with lower odds (OR = 0.27, 95% CI: 0.12 to 0.56, *p* < 0.001). Heel lift increased the likelihood of forward knees (OR = 9.20, 95% CI: 2.62 to 36.1, *p* < 0.001), whereas lacking depth reduced it (OR = 0.16, 95% CI: 0.05 to 0.42, *p* < 0.001). An incorrect knee path further increased the odds (OR = 2.96, 95% CI: 1.05 to 8.60, *p* = 0.040).

5. Predictors of Incorrect Knee Path

The pathway taken by the knee during the squat was recorded as “driving out in line with feet” (89.0%) and “incorrect knee path” (11.0%). The model yielded good discrimination (AUC = 0.722) and explained up to 15.8% of the variance (Nagelkerke R^2^). A lower skeletal muscle mass (kg) was significantly associated with increased odds of an incorrect knee path (OR = 0.87, 95% CI: 0.79 to 0.95, *p* = 0.004). A heel lift was a strong predictor as participants who lifted their heels during the squat had substantially higher odds of demonstrating an incorrect knee path than those keeping their heels grounded (OR = 7.09, 95% CI: 1.86 to 26.2, *p* = 0.003).

6. Predictors of Heel Lift

Heel lift was recorded as heel grounded (92.5%) or heel lift (7.5%). The model showed excellent discrimination (AUC = 0.904) and explained up to 42.8% of variance (Nagelkerke R^2^). Male participants had higher odds of heel lift compared to females (OR = 6.83, 95% CI: 1.65 to 36.2, *p* = 0.013). Neck pain was associated with lower odds of heel lift (OR = 0.05, 95% CI: 0.00 to 0.44, *p* = 0.023), whereas headaches increased the odds (OR = 7.36, 95% CI: 1.44 to 42.3, *p* = 0.018). Relative to butt-wink, neutral spine markedly increased odds (OR = 50.8, 95% CI: 7.14 to 1116, *p* = 0.001), whereas the absence of forward knee translation reduced the odds (OR = 0.07, 95% CI: 0.01 to 0.28, *p* < 0.001) for heel lift during the squat.

7. Predictors of Incorrect Stance

Feet position during the squat was recorded as shoulder width (71.5%) or non-shoulder width (28.5%), with the latter being performed by the aggregation of two categories: more than shoulder width (19.0%) and less than shoulder width (9.5%). The model showed fair discrimination (AUC = 0.677) and explained up to 10.5% of variance (Nagelkerke R^2^). Greater subcutaneous tissue thickness at Caliper Site 3 (OR = 1.04, 95% CI: 1.01 to 1.06, *p* = 0.009) and participants reporting neck pain were also more likely to position their feet outside the typical shoulder width alignment (OR = 2.25, 95% CI: 1.10 to 4.54, *p* = 0.024).

### 3.5. Predictors of a Perfect Squat

A perfect squat pattern was assumed to be observed when participants met no compensation in movement during the squat across all the observed variables. A total of 8% (n = 15) met this condition of a perfect squat. LASSO retained only the intercept term, indicating no strong linear associations between the demographic, anthropometric, postural or symptom variables and a perfect squat technique. Subsequently, a Firth penalized likelihood logistic regression model was fitted using variables identified from liberal univariable screening (*p* < 0.20), which ultimately included hip circumference, thoracic spine posture, shoulder posture, and neck pain. None of these predictors reached statistical significance (all *p* > 0.05). The direction of effects suggested that greater hip circumference was associated with slightly higher odds of achieving a perfect squat, whereas thoracic kyphosis, protracted shoulder posture, and neck pain were associated with lower odds. The model demonstrated acceptable discrimination (AUC = 0.758) but low explanatory power (Nagelkerke R^2^ = 0.189). These findings highlight the absence of strong individual predictors of a perfect squat within the available demographic, anthropometric, and symptom domains.

## 4. Discussion

### 4.1. Principal Findings

This study examined the multivariable associations between anthropometric characteristics, static posture, self-reported musculoskeletal and visceral symptoms, and visually assessed squat movement quality. Three principal findings emerged. First, body composition variables—particularly fat mass and skeletal muscle mass—were more consistently associated with discrete squat deviations than sex. Higher fat mass and greater skinfold thickness were associated with reduced squat depth, altered spinal alignment, and increased forward knee translation. Conversely, lower skeletal muscle mass significantly predicted incorrect knee tracking. Second, specific postural deviations influenced squat performance. A protracted shoulder posture was independently associated with limited squat depth, while thoracic kyphosis and shoulder protraction showed trends toward a reduced likelihood of achieving a “perfect” squat pattern. Third, selected self-reported symptoms were associated with compensatory movement strategies. Low back pain increased the likelihood of forward knee translation, headaches increased the odds of heel lift, and neck pain was associated with altered lower-limb positioning. However, no single demographic, anthropometric, or symptom variable independently predicted the achievement of a fully optimal squat pattern, suggesting that movement quality reflects multifactorial interactions rather than isolated predictors.

### 4.2. Body Composition and Squat Mechanics

The relationship between body composition and squat performance observed in this study aligns with the existing biomechanical literature. Higher fat mass was associated with reduced squat depth and altered spinal positioning. Increased adiposity likely modifies center-of-mass distribution and increases joint loading demands, requiring compensatory strategies such as forward knee displacement or spinal adjustments to maintain balance. These findings are consistent with previous research demonstrating associations between body composition and strength performance, kinematic adaptations, and movement efficiency during lower-limb tasks [8,9,22]. In contrast, skeletal muscle mass demonstrated a protective role. Lower muscle mass predicted incorrect knee path, suggesting insufficient neuromuscular control or strength capacity to stabilize frontal-plane knee alignment during descent and ascent. Muscle mass has previously been linked to force production capacity and movement stability [23], supporting the interpretation that neuromuscular capacity contributes substantially to squat quality. Importantly, sex differences in squat mechanics were minimal despite clear anthropometric differences between males and females. Although anatomical and strength differences between sexes are frequently reported in single-leg and bilateral squat analyses [24,25,26], our findings indicate that once body composition variables are accounted for, sex itself is not a dominant predictor of visual squat deviations. This suggests that modifiable characteristics such as muscle mass and fat distribution may be more clinically relevant than biological sex alone.

### 4.3. Static Posture and Movement Execution

Static postural alignment demonstrated meaningful associations with dynamic squat execution. Protracted shoulder posture increased the odds of limited squat depth, and thoracic kyphosis demonstrated a trend toward a reduced likelihood of optimal performance. The mechanistic explanation may relate to thoracolumbar alignment influencing trunk inclination and load distribution during the squat. Prior investigations have demonstrated that altered thoracolumbar positioning, including an anterior pelvic tilt, affects the spinal mechanics and balance control during loaded squatting tasks [27,28,29]. Excessive kyphosis or shoulder protraction may restrict thoracic extension, thereby altering the trunk–pelvis relationship and limiting depth. These findings reinforce the concept that static alignment may influence dynamic motor patterns, although it should be acknowledged that posture alone did not strongly predict overall squat perfection. Rather, posture appears to interact with anthropometric and neuromuscular factors to shape observable movement quality.

### 4.4. Self-Reported Symptoms and Compensatory Strategies

The presence of musculoskeletal symptoms influenced specific squat parameters. Low back pain increased the likelihood of forward knee translation, potentially reflecting protective unloading of the lumbar spine through anterior weight shift. Headaches were associated with heel lift, and neck pain influenced lower-limb positioning. Pain is known to alter motor control through protective adaptations and the redistribution of load [30,31]. Postural control impairments associated with headaches have also been described [32], which may explain the compensatory lower-limb strategies observed in this cohort. However, symptom presence did not strongly predict global squat quality, suggesting that symptoms influence discrete components rather than overall movement execution. It is noteworthy that visceral symptoms, although included in the assessment framework, demonstrated limited statistical influence on squat mechanics. This may reflect low prevalence rates within the sample or insufficient severity to influence dynamic performance.

### 4.5. The Multifactorial Nature of “Perfect” Squat Performance

Only 8% of participants achieved a fully “perfect” squat pattern. Multivariable modeling did not identify strong independent predictors of this outcome. This finding suggests that optimal movement quality is unlikely to be determined by single anthropometric, postural, or symptom variables. Instead, it appears to reflect the integrated interaction of neuromuscular control, body composition, alignment, and possibly unmeasured variables such as training experience and motor learning history. The absence of strong predictors reinforces the complexity of human movement and cautions against reductionist interpretations of squat performance based solely on isolated characteristics.

### 4.6. Clinical and Applied Implications

From a clinical perspective, these findings suggest that practitioners should prioritize modifiable characteristics when assessing squat performance. Screening should include a) body composition profiling, particularly fat mass and skeletal muscle mass; b) thoracolumbar and shoulder alignment assessment; and c) symptom history, particularly low back pain and cervical-related complaints. Rather than attributing squat deviations primarily to sex differences, clinicians and kinesiologists may benefit from targeting neuromuscular strengthening and alignment correction strategies. In applied contexts, particularly within digital or camera-based screening systems, algorithms may improve the predictive accuracy by incorporating anthropometric and posture-derived inputs rather than relying solely on joint angle estimation.

### 4.7. Strengths and Limitations

This study employed multivariable penalized regression modeling, reducing dimensionality bias and limiting overfitting in predictor selection. The integration of anthropometric, postural, and symptom variables within a single analytical framework represents a novel contribution to applied squat screening research. However, several limitations must be acknowledged. The cross-sectional design precludes causal inference. Prior resistance training experience and motor learning history were not formally quantified and may have influenced movement execution. Visual classification, although reflective of real-world screening practice, lacks the precision of three-dimensional motion capture systems. Additionally, the relatively young and predominantly White cohort may limit the generalizability.

### 4.8. Future Directions

Future investigations should incorporate quantified training history and physical activity levels; longitudinal designs to evaluate the modification of predictors over time; and validation of visual classification findings against instrumented motion capture. Understanding how modifiable anthropometric and postural characteristics interact with neuromuscular capacity may enhance both clinical screening and digital movement assessment technologies.

## 5. Conclusions

This study identified clear gender differences in anthropometric profiles, posture, and symptom prevalence, while most squat mechanics parameters were comparable between men and women. Women exhibited superior shoulder and thoracic posture but reported higher rates of neck pain, headaches, and gastrointestinal symptoms. Key predictors of suboptimal squat mechanics included lower skeletal muscle mass, higher fat mass and skinfold thickness, and specific lower-limb movement patterns such as heel lift and limited forward knee translation. Greater hip circumference and appropriate lower-limb mechanics were protective against cervical hyperextension and non-neutral spinal alignment. Although several anthropometric and biomechanical factors influenced discrete aspects of squat performance, a “perfect” squat pattern was rare and not strongly predicted by any single variable, suggesting that optimal movement quality reflects a multifactorial interplay of posture, body composition, and motor control rather than isolated physical characteristics. Squat mechanics are more strongly influenced by body composition, posture, and symptoms than by gender. These factors should be prioritized by kinesiologists and physiotherapists when designing individualized training and rehabilitation programs to optimize squat performance and reduce injury risk.

## Figures and Tables

**Table 1:** Demographic characteristics across gender against self-reported symptoms.

Variables	Overall N = 200 ^1^	Female N = 115 ^1^	Male N = 85 ^1^	*p*-Value ^2^
Age	26.0 (21.0, 35.0)	26.0 (21.0, 38.0)	26.0 (21.0, 34.0)	0.51
Height (cm)	168.5 (9.0)	163.6 (6.9)	175.3 (6.8)	<0.001
Weight (kg)	67.4 (57.8, 78.0)	61.1 (53.8, 68.2)	77.2 (68.1, 83.6)	<0.001
Race				0.46
African American	4.0 (2.0%)	2.0 (1.7%)	2.0 (2.4%)	
Asian	2.0 (1.0%)	0.0 (0.0%)	2.0 (2.4%)	
Hispanic	10.0 (5.0%)	6.0 (5.2%)	4.0 (4.7%)	
North African	2.0 (1.0%)	2.0 (1.7%)	0.0 (0.0%)	
White	182.0 (91.0%)	105.0 (91.3%)	77.0 (90.6%)	
Hip Circumference (cm)	92.0 (86.5, 100.0)	91.5 (85.0, 100.0)	94.0 (87.0, 100.0)	0.32
Waist Circumference (cm)	78.0 (72.3, 87.8)	74.0 (69.0, 81.0)	85.0 (78.0, 91.0)	<0.001
FM BCA (%)	22.1 (15.1, 29.7)	26.8 (21.1, 33.7)	16.0 (11.3, 20.0)	<0.001
Fat-Free Mass (kg)	51.1 (42.9, 62.7)	44.0 (39.6, 47.8)	64.2 (57.8, 68.8)	<0.001
Skeletal Muscle Mass (kg)	25.0 (20.4, 32.2)	21.0 (18.4, 23.0)	32.2 (29.4, 35.7)	<0.001
Caliper Site 1 (mm)	13.0 (6.6, 18.5)	15.0 (9.2, 20.2)	7.3 (5.3, 15.0)	<0.001
Caliper Site 2 (mm)	14.0 (10.0, 22.0)	14.0 (10.0, 22.0)	14.4 (9.9, 22.7)	0.90
Caliper Site 3 (mm)	18.6 (12.0, 27.0)	21.0 (12.2, 31.7)	16.0 (10.8, 21.9)	0.001
Body Density	1.1 (1.0, 1.1)	1.1 (1.0, 1.1)	1.1 (1.1, 1.1)	<0.001
FM Caliper (%)	16.9 (10.8, 24.9)	19.4 (14.9, 28.5)	11.7 (8.4, 16.9)	<0.001
Muscle Mass %	0.8 (0.7, 0.8)	0.7 (0.7, 0.8)	0.8 (0.8, 0.9)	<0.001
Fat Mass (kg)	13.8 (9.9, 20.7)	15.2 (11.7, 22.9)	11.9 (8.2, 15.0)	<0.001
Neck Pain	49.0 (24.5%)	38.0 (33.0%)	11.0 (12.9%)	0.001
Low Back Pain	66.0 (33.0%)	44.0 (38.3%)	22.0 (25.9%)	0.066
Headaches	33.0 (16.5%)	29.0 (25.2%)	4.0 (4.7%)	<0.001
Hypertension	5.0 (2.5%)	3.0 (2.6%)	2.0 (2.4%)	>0.99
Heartburn	18.0 (9.0%)	16.0 (13.9%)	2.0 (2.4%)	0.005
Constipation	18.0 (9.0%)	15.0 (13.0%)	3.0 (3.5%)	0.020
History of Hernia	7.0 (3.5%)	2.0 (1.7%)	5.0 (5.9%)	0.14
Incontinence	3.0 (1.5%)	3.0 (2.6%)	0.0 (0.0%)	0.26

^1^ Median (Q1, Q3); n (%); Mean (SD); ^2^ Wilcoxon rank sum test; Fisher’s exact test; Pearson’s Chi-squared test.

**Table 2:** Demographic characteristics across gender against posture.

Variables	Overall N = 200 ^1^	Female N = 115 ^1^	Male N = 85 ^1^	*p*-Value ^2^
Head Posture				0.32
Forward Head	120.0 (61.9%)	72.0 (64.9%)	48.0 (57.8%)	
Good Posture	74.0 (38.1%)	39.0 (35.1%)	35.0 (42.2%)	
Cervical Spine Posture				0.72
Forward Head	122.0 (62.9%)	71.0 (64.0%)	51.0 (61.4%)	
Good Posture	72.0 (37.1%)	40.0 (36.0%)	32.0 (38.6%)	
Shoulder Posture				<0.001
Good Posture	123.0 (63.1%)	83.0 (74.1%)	40.0 (48.2%)	
Protracted Shoulders	72.0 (36.9%)	29.0 (25.9%)	43.0 (51.8%)	
Thoracic Spine Posture				<0.001
Good Posture	110.0 (57.3%)	83.0 (75.5%)	27.0 (32.9%)	
Thoracic Kyphosis	82.0 (42.7%)	27.0 (24.5%)	55.0 (67.1%)	
Lumbar Spine Posture				0.40
Lumbar Hyperlordosis	85.0 (44.3%)	52.0 (46.8%)	33.0 (40.7%)	
Lumbar Hypolordosis	8.0 (4.2%)	3.0 (2.7%)	5.0 (6.2%)	
Neutral Spine	99.0 (51.6%)	56.0 (50.5%)	43.0 (53.1%)	
Pelvic Tilt Posture				0.57
Neither ASIS/PSIS	98.0 (51.3%)	55.0 (49.5%)	43.0 (53.8%)	
Pelvic tilt	93.0 (48.7%)	56.0 (50.5%)	37.0 (46.3%)	

^1^ n (%); ^2^ Fisher’s exact test; Pearson’s Chi-squared test.

**Table 3:** Demographic characteristics across gender against squat characteristics.

Variables	Overall N = 200 ^1^	Female N = 115 ^1^	Male N = 85 ^1^	*p*-Value ^2^
Squat Mechanics: Spine				0.13
Butt-Wink	80.0 (40.0%)	40.0 (34.8%)	40.0 (47.1%)	
Lumbar Hyperextension	14.0 (7.0%)	7.0 (6.1%)	7.0 (8.2%)	
Neutral	106.0 (53.0%)	68.0 (59.1%)	38.0 (44.7%)	
Squat Mechanics: Neck				0.50
Cervical Hyperextension	91.0 (45.5%)	50.0 (43.5%)	41.0 (48.2%)	
Neutral	109.0 (54.5%)	65.0 (56.5%)	44.0 (51.8%)	
Squat Mechanics: Feet				0.15
Heel Grounded	185.0 (92.5%)	109.0 (94.8%)	76.0 (89.4%)	
Heel Lift	15.0 (7.5%)	6.0 (5.2%)	9.0 (10.6%)	
Squat Mechanics: ROM				0.68
Good Depth	150.0 (75.0%)	85.0 (73.9%)	65.0 (76.5%)	
Lacking Depth	50.0 (25.0%)	30.0 (26.1%)	20.0 (23.5%)	
Squat Mechanics: Forward Knees				0.52
Forward Knees	71.0 (35.5%)	43.0 (37.4%)	28.0 (32.9%)	
No Forward Knees	129.0 (64.5%)	72.0 (62.6%)	57.0 (67.1%)	
Squat Mechanics: Feet Position				0.25
Less Than Shoulder Width	19.0 (9.5%)	13.0 (11.3%)	6.0 (7.1%)	
More Than Shoulder Width	38.0 (19.0%)	25.0 (21.7%)	13.0 (15.3%)	
Shoulder Width	143.0 (71.5%)	77.0 (67.0%)	66.0 (77.6%)	
Squat Mechanics: Feet Rotation				0.002
Excessive Outward Rotation	66.0 (33.0%)	29.0 (25.2%)	37.0 (43.5%)	
Inward Rotation	7.0 (3.5%)	7.0 (6.1%)	0.0 (0.0%)	
Slight Outward Rotation	127.0 (63.5%)	79.0 (68.7%)	48.0 (56.5%)	
Squat Mechanics: Knees Path				0.047
Drive Out in Line with Feet	178.0 (89.0%)	98.0 (85.2%)	80.0 (94.1%)	
Incorrect Knee Path	22.0 (11.0%)	17.0 (14.8%)	5.0 (5.9%)	

^1^ n (%); ^2^ Fisher’s exact test; Pearson’s Chi-squared test.

## Data Availability

The original contributions presented in this study are included in the article. Further inquiries can be directed to the corresponding author(s).

## Abbreviations

The following abbreviations are used in this manuscript:

BMI	Body Mass Index
LBP	Low Back Pain
BCA	Body Composition Analysis
ROM	Range of Movement

## Appendix A

### Appendix A.1

**Logistic Regression Model—Predicting Cervical Hyperextension** **% Participants with Outcome = 45.5%** **McFadden R^2^ = 0.054, Cox–Snell R^2^ = 0.071, Nagelkerke R^2^ = 0.095,** **AUC = 0.658**			
**Characteristic**	**OR**	**95% CI**	***p*** **-Value**
(Intercept)	20.7	1.61, 314	0.024
Hip Circumference (cm)	0.96	0.94, 0.99	0.009
Forward Knees			
Forward Knees	—	—	
No Forward Knees	2.03	1.11, 3.72	0.021
Null Deviance	276		
Null df	199		
Log-likelihood	−130		
AIC	267		
BIC	277		
Deviance	261		
Residual df	197		
No. Obs.	200		
Abbreviations: CI = confidence interval, OR = odds ratio.			

### Appendix A.2

**Logistic Regression Model—Predicting Non-Neutral Spine ** **% Participants with Outcome = 47%** **McFadden R^2^ = 0.196, Cox–Snell R^2^ = 0.237, Nagelkerke R^2^ = 0.317,** **AUC = 0.787**			
**Characteristic**	**OR**	**95% CI**	***p*** **-Value**
(Intercept)	5.89	2.18, 17.1	<0.001
FM BCA (%)	0.90	0.86, 0.94	<0.001
Caliper Site 2 (mm)	1.04	1.00, 1.09	0.041
Caliper Site 3 (mm)	1.04	1.00, 1.08	0.032
Feet			
Heel Grounded	—	—	
Heel Lift	0.07	0.01, 0.31	0.002
Forward Knees			
Forward Knees	—	—	
No Forward Knees	0.18	0.08, 0.36	<0.001
Null Deviance	277		
Null df	199		
Log-likelihood	−111		
AIC	234		
BIC	254		
Deviance	222		
Residual df	194		
No. Obs.	200		
Abbreviations: CI = confidence interval, OR = odds ratio.			

### Appendix A.3

**Logistic Regression Model—Predicting Lacking Depth (ROM)** **Participants with Outcome = 25%** **McFadden R^2^ = 0.236, Cox–Snell R^2^ = 0.233, Nagelkerke R^2^ = 0.345,** **AUC = 0.816**			
**Characteristic**	**OR**	**95% CI**	***p*** **-Value**
(Intercept)	0.00	0.00, 0.01	<0.001
Caliper Site 2 (mm)	1.06	1.02, 1.11	0.006
Fat Mass (kg)	1.06	1.00, 1.11	0.033
Shoulders			
Good Posture	—	—	
Protracted Shoulders	2.66	1.16, 6.31	0.022
Spine			
Butt-Wink	—	—	
Lumbar Hyperextension	24.1	4.92, 135	<0.001
Neutral	5.33	2.07, 15.4	0.001
Forward Knees			
Forward Knees	—	—	
No Forward Knees	4.19	1.70, 11.5	0.003
Knees Path			
Drive Out in Line with Feet	—	—	
Incorrect Knee Path	5.53	1.80, 17.6	0.003
Null Deviance	225		
Null df	199		
Log-likelihood	−86.0		
AIC	188		
BIC	214		
Deviance	172		
Residual df	192		
No. Obs.	200		
Abbreviations: CI = confidence interval, OR = odds ratio.			

### Appendix A.4

**Logistic Regression Model—Predicting Forward Knees** **% Participants with Outcome = 35.5%** **McFadden R^2^ = 0.192, Cox–Snell R^2^ = 0.221, Nagelkerke R^2^ = 0.304** **AUC = 0.784**			
**Characteristic**	**OR**	**95% CI**	***p*** **-Value**
(Intercept)	0.27	0.11, 0.63	0.003
FM BCA (%)	1.05	1.01, 1.09	0.010
Low Back Pain			
No	—	—	
Yes	2.04	1.01, 4.18	0.047
Spine			
Butt-Wink	—	—	
Lumbar Hyperextension	4.44	1.17, 19.7	0.036
Neutral	0.27	0.12, 0.56	<0.001
Feet			
Heel Grounded	—	—	
Heel Lift	9.20	2.62, 36.1	<0.001
ROM			
Good Depth	—	—	
Lacking Depth	0.16	0.05, 0.42	<0.001
Knees Path			
Drive Out in Line with Feet	—	—	
Incorrect Knee Path	2.96	1.05, 8.60	0.040
Null Deviance	260		
Null df	199		
Log-likelihood	−105		
AIC	226		
BIC	253		
Deviance	210		
Residual df	192		
No. Obs.	200		
Abbreviations: CI = confidence interval, OR = odds ratio.			

### Appendix A.5

**Logistic Regression Model—Predicting Incorrect Knee Path** **% Participants with Outcome = 11%** **Cox–Snell R^2^ = 0.079, Nagelkerke R^2^ = 0.158, AUC = 0.722**			
**Characteristic**	**OR**	**95% CI**	***p*** **-Value**
(Intercept)	2.52	0.33, 22.5	0.4
Skeletal Muscle Mass (kg)	0.87	0.79, 0.95	0.004
Feet			
Heel Grounded	—	—	
Heel Lift	7.09	1.86, 26.2	0.003
Null Deviance	139		
Null df	199		
Log-likelihood	−61.1		
AIC	128		
BIC	138		
Deviance	122		
Residual df	197		
No. Obs.	200		
Abbreviations: CI = confidence interval, OR = odds ratio.			

### Appendix A.6

**Logistic Regression Model—Predicting Heel Lift ** **% Participants with Outcome = 7.5%** **McFadden R^2^ = 0.365, Cox–Snell R^2^ = 0.177, Nagelkerke R^2^ = 0.428,** **AUC = 0.904**			
**Characteristic**	**OR**	**95% CI**	***p*** **-Value**
(Intercept)	0.01	0.00, 0.05	<0.001
Gender			
Female	—	—	
Male	6.83	1.65, 36.2	0.013
Neck Pain			
No	—	—	
Yes	0.05	0.00, 0.44	0.023
Headaches			
No	—	—	
Yes	7.36	1.44, 42.3	0.018
Spine			
Butt-Wink	—	—	
Lumbar Hyperextension	6.76	0.22, 210	0.2
Neutral	50.8	7.14, 1116	0.001
Forward Knees			
Forward Knees	—	—	
No Forward Knees	0.07	0.01, 0.28	<0.001
Null Deviance	107		
Null df	199		
Log-likelihood	−33.8		
AIC	81.6		
BIC	105		
Deviance	67.6		
Residual df	193		
No. Obs.	200		
Abbreviations: CI = confidence interval, OR = odds ratio.			

### Appendix A.7

**Logistic Regression Model—Predicting Non-Shoulder Width (Feet) ** **% Participants with Outcome = 28.5%** **McFadden R^2^ = 0.064, Cox–Snell R^2^ = 0.074, Nagelkerke R^2^ = 0.105,** **AUC = 0.677**			
**Characteristic**	**OR**	**95% CI**	***p*** **-Value**
(Intercept)	0.15	0.07, 0.28	<0.001
Caliper Site 3 (mm)	1.04	1.01, 1.06	0.009
Neck Pain			
No	—	—	
Yes	2.25	1.10, 4.54	0.024
Null Deviance	239		
Null df	199		
Log-likelihood	−112		
AIC	230		
BIC	240		
Deviance	224		
Residual df	197		
No. Obs.	200		
Abbreviations: CI = confidence interval, OR = odds ratio.			

### Appendix A.8

**Logistic Regression Model—Predicting Non-Slight Outward Rotation (Feet) ** **% Participants with Outcome = 36.5%** **McFadden R^2^ = 0.047, Cox–Snell R^2^ = 0.06, Nagelkerke R^2^ = 0.082,** **AUC = 0.615**			
**Characteristic**	**OR**	**95% CI**	***p*** **-Value**
(Intercept)	0.27	0.08, 0.73	0.019
Feet Position			
Less Than Shoulder Width	—	—	—
More Than Shoulder Width	5.75	1.71, 23.3	0.007
Shoulder Width	1.78	0.61, 6.50	0.3
Null deviance	262		
Null df	199		
Log-likelihood	−125		
AIC	256		
BIC	266		
Deviance	250		
Residual df	197		
No. Obs.	200		
Abbreviations: CI = confidence interval, OR = odds ratio.			

### Appendix A.9

**Firth Penalized Likelihood Logistic Regression Model—** **Predicting Perfect Squat Technique** **% Participants with Outcome = 8.0%** **McFadden R^2^ = 0.159, Cox–Snell R^2^ = 0.065, Nagelkerke R^2^ = 0.189,** **AUC = 0.758**			
**Characteristic**	**OR**	**95% CI**	***p*** **-Value**
(Intercept)	0.029	0.001, 0.121	0.000
Hip Circumference (cm)	1.023	0.967, 1.079	0.418
Thoracic Spine			
Good Posture	—	—	—
Thoracic Kyphosis	0.339	0.060, 1.307	0.122
Neck pain			
No	—	—	—
Yes	0.290	0.031, 1.254	0.105
Shoulder Posture			
Good Posture	—	—	—
Protracted Shoulders	0.524	0.094, 2.035	0.371
AIC	83.2		
BIC	103.0		
No. Obs.	200		
Abbreviations: CI = confidence interval, OR = odds ratio.			
